# The N6-methyladenosine RNA epigenetic modification modulates the amplification of coxsackievirus B1 in human pancreatic beta cells

**DOI:** 10.3389/fmicb.2024.1501061

**Published:** 2024-12-18

**Authors:** Maressa Fernandes Bonfim, Camille Aitchedji, Flore Van Goethem, Lionel Sauvage, Thibault Poinsot, Emilie Calonne, Rachel Deplus, François Fuks, Decio L. Eizirik, Anne Op de Beeck

**Affiliations:** ^1^ULB Center for Diabetes Research, Medical Faculty, Université Libre de Bruxelles, Brussels, Belgium; ^2^The Laboratory of Cancer Epigenetics, Medical Faculty, Université Libre de Bruxelles, Brussels, Belgium; ^3^Laboratory of Microbiology, European Plotkin Institute for Vaccinology, Université Libre de Bruxelles, Brussels, Belgium

**Keywords:** m6A, coxsackievirus, pancreatic beta cell, type 1 diabetes, epigenetic regulation

## Abstract

Type 1 diabetes (T1D) is characterized by a prolonged autoimmune attack resulting in the massive loss of insulin-producing beta cells. The initiation and progression of T1D depends on a complex interaction between genetic, immunological and environmental factors. Epidemiological, experimental and clinical evidence suggest a link between viral infections, particularly Coxsackievirus type B (CVB), and T1D development. Specifically, infections by the CVB serotype 1 (CVB1) contribute to the triggering of autoimmunity against beta cells in genetically predisposed individuals, and prolonged and probably non-lytic infections by CVB are associated with the development of T1D. However, the molecular mechanisms underlying CVB1 replication and establishing persistent infections in human pancreatic beta cells remain poorly understood. Here we show that the N6-methyladenosine (m6A) RNA epigenetic modification machinery regulates CVB1 amplification in the human beta cells. Using small interfering RNA (siRNA) targeting m6A writers and erasers, we observed that downregulation of m6A writers increases CVB1 amplification, while the downregulation of m6A erasers decreases it. Notably, the inhibition of Fat Mass and Obesity-associated protein (FTO), a key m6A eraser, reduced by 95% the production of infectious CVB1 in both human insulin-producing EndoC-βH1 cells and in induced pluripotent stem cell (iPSC)-derived islets. The FTO inhibitor reduced CVB1 expression within 6 h post-infection, suggesting a direct regulation of the CVB1 genome by m6A modification. Furthermore, in the absence of viral replication, FTO inhibition also decreased the translation of the incoming CVB1 genome, indicating that m6A plays a critical role in the initial stages of viral RNA translation. In addition, modulation of the m6A machinery affected the type I interferon response after poly-IC transfection, a mimic of RNA virus replication, but did not affect the cellular antiviral response in CVB1-infected cells. Altogether, these observations suggest that m6A directly affects CVB1 production. Our study provides the first evidence that the m6A epigenetic modification machinery controls CVB amplification in human pancreatic beta cells. This suggests that the m6A machinery is a potential target to control CVB infection in T1D and raises the possibility of an epigenetic control in the establishment of persistent CVB infections observed in the pancreas in individuals with type 1 diabetes.

## 1 Introduction

Type 1 diabetes (T1D) results from an autoimmune attack and massive loss of functional insulin-producing beta cells in the pancreas. Predisposition to T1D is influenced by HLA class II and non-HLA genes, but genetic susceptibility does not explain the increasing annual incidence rate of T1D (Norris et al., 2020). Epidemiological, clinical and experimental studies strongly support the involvement of enteroviruses in the development of T1D (Patterson et al., 2019; Gregory et al., 2022). Among enterovirus infections, coxsackievirus B (CVB) are the prime suspect as a diabetogenic environmental triggers (Nekoua et al., 2022). CVBs have a tropism for beta cells and autopsy or biopsy samples of the pancreas of patients with newly diagnosed T1DM suggest the presence of persistent non lytic enterovirus infections (Richardson et al., 2009; Apaolaza et al., 2021). Prospective studies in children genetically at risk to develop T1D reveal that the persistence of enteroviruses, particularly CVB, is associated with the onset of islet autoimmunity (Honkanen et al., 2017; Kim et al., 2019; Oikarinen et al., 2012; Vehik et al., 2019) and that the infection with the CVB serotype 1 (CVB1) is strongly associated with the initiation of insulin-driven autoimmunity (Laitinen et al., 2014; Sioofy-Khojine et al., 2018). Despite this accumulating information, the molecular mechanisms of CVB1 replication in human pancreatic beta cells remain poorly understood.

As a single-stranded positive-sense RNA virus, the CVB genome can undergo various epigenetic modifications that affect its amplification. More than 150 types of epigenetic modifications on RNA have been identified so far (Boccaletto et al., 2018). The m6A modification is one of the most abundant and is found in the consensus motif RRm6ACH ([G/A/U][G/A]m6AC[U/A/C]) (Liu and Pan, 2015). This modification is detected on mRNA, rRNA, microRNA but also on viral RNA. The M6A modification affects mRNA stability, splicing, export from the nucleus and translation (Wang et al., 2014; Liu and Pan, 2015; Wang et al., 2015). When present in RNA viral genomes, m6A impacts the viral cycle (Brocard et al., 2017; Horner and Reaves, 2024). M6A is added by a methyltransferase complex (writers) which includes METTL3, METTL14 and WTAP and is removed by the demethylases FTO and ALKBH5 (erasers). The YTH domain family of proteins and other “readers” recognize and bind to the m6A-modified sites and directly regulate the posttranscriptional functions of the modified mRNA (Jiang et al., 2021). Readers also bind to m6A-modified viral RNA and impact their viral cycle at different stages of the cycle (Zhong et al., 2024; Ge et al., 2023; Yang et al., 2023). Against this background, the goal of the present study is to understand how m6A modification affects the amplification of CVB1 in human pancreatic beta cells.

We observed that the cellular machinery responsible for the m6A modification of RNA modulates the amplification of CVB1 in the human pancreatic beta cell line EndoC-βH1 and in human iPSC derived islets. These observations suggest that m6A directly affects CVB1 replication.

## 2 Materials and methods

### 2.1 Culture of EndoC-βH1 cells

The human pancreatic beta cell line, EndoC-βH1 (Ravassard et al., 2011), was kindly provided by Dr. R. Scharfmann (Cochin Institute, Université Paris Descartes, Paris, France). These cells were cultured in DMEM containing 5.6 mmoL/L glucose (Gibco, Thermo Fisher Scientific, Waltham, MA, United States), supplemented with 2% fatty acid-free BSA (Roche, Basel, Switzerland), 50 μmoL/L 2-mercaptoethanol (Sigma-Aldrich, St. Louis, MO, United States), 10 mmoL/L nicotinamide (Calbiochem, Darmstadt, Germany), 5.5 μg/mL transferrin, 6.7 ng/mL sodium selenite (both from Sigma-Aldrich), and 100 U/mL penicillin +100 μg/mL streptomycin (Lonza, Leusden, Netherlands). The cells were plated on Matrigel–fibronectin-coated plates, as described previously (Ravassard et al., 2011). To ensure the cells were free from mycoplasma contamination, regular monthly testing was performed using the MycoAlert Mycoplasma Detection kit (Lonza, Basel, Switzerland). EndoC-βH1 cells were utilized up to passage 70, starting from frozen stocks at passage 45–55. Typically, two independent cultures from different batches and passages, maintained by different researchers, were available simultaneously in the lab. Experiments were conducted over several weeks using either culture independently or in parallel, to increase the number of independent replicates.

### 2.2 Culture and differentiation of induced pluripotent stem cells into islet-like cells

In this study the iPSC line 1023A, provided by DM Egli (Columbia University, United States), was used. Ethical approval for the differentiation of iPSCs into islet-like cells was granted by the Ethics Committee of Erasmus Hospital, Université Libre de Bruxelles (reference P2019/498). iPSCs were cultured on Matrigel-coated plates (Corning, NY, United States) using E8 medium (Invitrogen Life Technologies, Paisley, UK) and passaged twice weekly with 0.5 mmoL/L EDTA (Invitrogen Life Technologies). Regular assessment of iPSC quality and pluripotency was carried out via immunocytochemical staining of pluripotency markers, as previously described (Lytrivi et al., 2021). The iPSCs were differentiated into beta cells using a seven-step protocol developed by our research group (Cosentino et al., 2018; Fantuzzi et al., 2022). After differentiation, the cell aggregates were dissociated, seeded onto Matrigel-coated plates, and maintained in HAM’s F-10 medium (Thermo Fisher Scientific, Waltham, MA, United States) supplemented with 2% fatty acid-free BSA (Roche, Basel, Switzerland), 2 mmoL/L GlutaMAX (Thermo Fisher Scientific), and 100 U/mL penicillin–streptomycin (Thermo Fisher Scientific) in preparation for CVB1 infection. By the end of stage 7, the resulting iPSC-derived beta-cell-like population included approximately 49 ± 3% insulin-positive cells, 6 ± 2% glucagon-positive cells, 2 ± 0.5% somatostatin-positive cells, 1.67 ± 0.2% insulin-glucagon double-positive cells, and 0.72 ± 0.2% insulin-somatostatin double-positive cells (*n* = 4).

### 2.3 Viral infection and titration

The prototype strain of enterovirus, CVB1/Conn-5, was acquired from the American Type Culture Collection (ATCC, Old Town Manassas, VA, United States). This virus was propagated in green monkey kidney (GMK) cells. EndoC-βH1 cells and iPSC derived-islets were infected with CVB1 at indicated multiplicity of infection (M.O.I.) in EndoC-βH1 or iPSC media without BSA and containing 2% FBS. After a 2-h adsorption period at 37°C, the inoculum was removed and complete culture medium was then added, allowing the virus to replicate for the specified durations. Viral production in the medium of infected cells was assessed by titration using endpoint dilutions in microwell cultures of GMK cells. After 6 days, cytopathic effects were observed via microscopy, and 50% tissue infection dose titers were calculated using the Kärber formula (Lennette and Schmidt, 1969). Relative viral titers were expressed as the ratio between viral titers in the treated and control conditions.

### 2.4 Cell treatment and small RNA interference

EndoC-βH1 cells and iPSC-derived islets were treated with Meclofenamic Acid (MA, M4531, Sigma-Aldrich) at concentrations of 50 μM and 25 μM, respectively, at the time of CVB1 infection, for the specified durations. In some experiments EndoC-βH1 cells were additionally treated with the nucleoside analog Gemcitabine hydrochloride (Gem, G6423, Sigma-Aldrich) at the indicated concentrations simultaneously with CVB1 infection.

For gene silencing, EndoC-βH1 cells were transfected with a combination of three siRNAs targeting METTL3 and one or two specific siRNAs targeting WTAP, FTO, and ALKBH5 for two consecutive days, with a final concentration of 30 nmol/L, following previously established protocols (Santin et al., 2016). Allstars Negative Control siRNA (Qiagen, Venlo, The Netherlands) was used as a negative control (siCTRL). The sequences of the siRNAs used in this study are provided in Supplementary Table S1.

Transfection of EndoC-βH1 cells with the synthetic double-stranded RNA (dsRNA) analog polyinosinic-polycytidylic acid (PIC, P0913, Sigma-Aldrich) was performed for 8 h at a final concentration of 1 μg/mL using Lipofectamine 2000 (Invitrogen, Carlsbad, CA, United States).

### 2.5 Assessment of cell viability and cytopathogenic effect

The percentage of viable, apoptotic, and cytopathogenic cells was further determined after staining with the DNA-binding dyes Hoechst 33342 (10 μg/mL) (Sigma-Aldrich) and propidium iodide (10 μg/mL) (Sigma-Aldrich). A minimum of 500 cells per experimental condition were counted by two independent observers, one of whom was blinded to sample identity. The inter-observer agreement remained above 90% throughout the analysis. Results are expressed as the percentage of apoptosis, calculated using the formula: (number of apoptotic cells / total number of cells) × 100.

### 2.6 RNA extraction and quantitative reverse transcription PCR (RT-qPCR)

Polyadenylated mRNA was isolated from cells using the Dynabeads mRNA DIRECT Kit (Invitrogen, Carlsbad, CA, United States) according to the manufacturer’s instructions. Following isolation, the mRNA was reverse transcribed into cDNA using the Reverse Transcriptase Core Kit (Eurogentec, Liège, Belgium). Quantitative PCR (qPCR) was performed with SYBR Green Supermix (Bio-Rad, Hercules, CA, United States) and ran in the CFX Connect Real-Time PCR Detection System (Bio-Rad). The amplicon levels were quantified using the standard curve method (Overbergh et al., 1999). Gene expression levels were normalized using the geometric mean of ACTB and VAPA as described (Alvelos et al., 2021). Primer sequences used in these experiments are detailed in Supplementary Table S2.

### 2.7 Protein extraction and Western blot analysis

Cells were washed with cold PBS and lysed in Laemmli Buffer (60 mM Tris–HCl pH 6.8, 10% glycerol, 1.5% dithiothreitol, 1.5% 2-mercaptoethanol, 2% SDS, and 0.005% bromophenol blue). For immunoblot analysis, membranes were incubated overnight with specific antibodies as detailed in Supplementary Table S3. After primary antibody incubation, membranes were exposed to secondary peroxidase-conjugated antibodies for 1 h at room temperature. Immunoreactive bands were visualized using the SuperSignal West Femto chemiluminescent substrate (Thermo Scientific) and detected with a ChemiDoc XRS+ system (Bio-Rad). The intensity of the bands was quantified using Image Lab software (Bio-Rad). The intensity of the bands was quantified using the Volume Tools in Bio-Rad’s Image Lab 6.1 software (Hercules, California, United States). Western blot bands were manually selected, and signal intensity was measured through volume integration with background subtraction.

### 2.8 Statistical analysis

Data are presented as the mean ± SEM, with individual data points shown for independent experiments. Normality was assessed using a normality test to determine Gaussian distribution. When data followed a normal distribution, group differences were analyzed using two-tailed unpaired Student *t* test or repeated measures one-way ANOVA, followed by Bonferroni *post hoc* tests. For non-normal distributions, the non-parametric Kruskal-Wallis test was applied as the equivalent to one-way ANOVA. Statistical significance was defined as *p* < 0.05 and the analyses were conducted using GraphPad Prism 8.0.1 (Dotmatics, Boston, MA, United States).

## 3 Results

### 3.1 The m6A writers complex reduce CVB1 amplification in EndoC-βH1 cells

The methyltransferase METTL3 is responsible for more than 95% of m6A in mRNA (Poh et al., 2022). To investigate the role of m6A machinery in the amplification of coxsackievirus in pancreatic human beta cells, we silenced METTL3 in the EndoC-βH1 cell line. Three different siRNA were tested leading to around 50% silencing efficacy (Supplementary Figures S1A,B) even after two rounds of successive silencing. METTL3 is challenging to silence since cells produce alternatively spliced METTL3 transcript isoforms and altered METTL3 proteins in KO cell lines (Poh et al., 2022). To improve METTL3 silencing efficiency, we pooled siRNAs targeting different METTL3 exons and achieved around 70% reduction in METTL3 protein synthesis (Supplementary Figure S1B), without increasing cell death (Supplementary Figure S1C).

Our objective was to detect the potential impact of silencing METTL3 on CVB1 amplification, so we set up infection conditions to avoid superinfection and excessive cell death. While the multiplicity of infection (M.O.I.) 1 and 0.1 led to 100 and 80% of dead cells respectively, the M.O.I. 0.01 led to a 50% cytopathogenic effect (CPE) in EndoC-βH1 cells 24 h post infection (Supplementary Figure S2A), and to the production of mean viral titers of 6.00E+06 TCID50/ml (Supplementary Figure S2B). This viral concentration is not saturating and can be used to detect changes in viral production. The M.O.I. of 0.01 was thus chosen for subsequent experiments.

**Figure 1:**
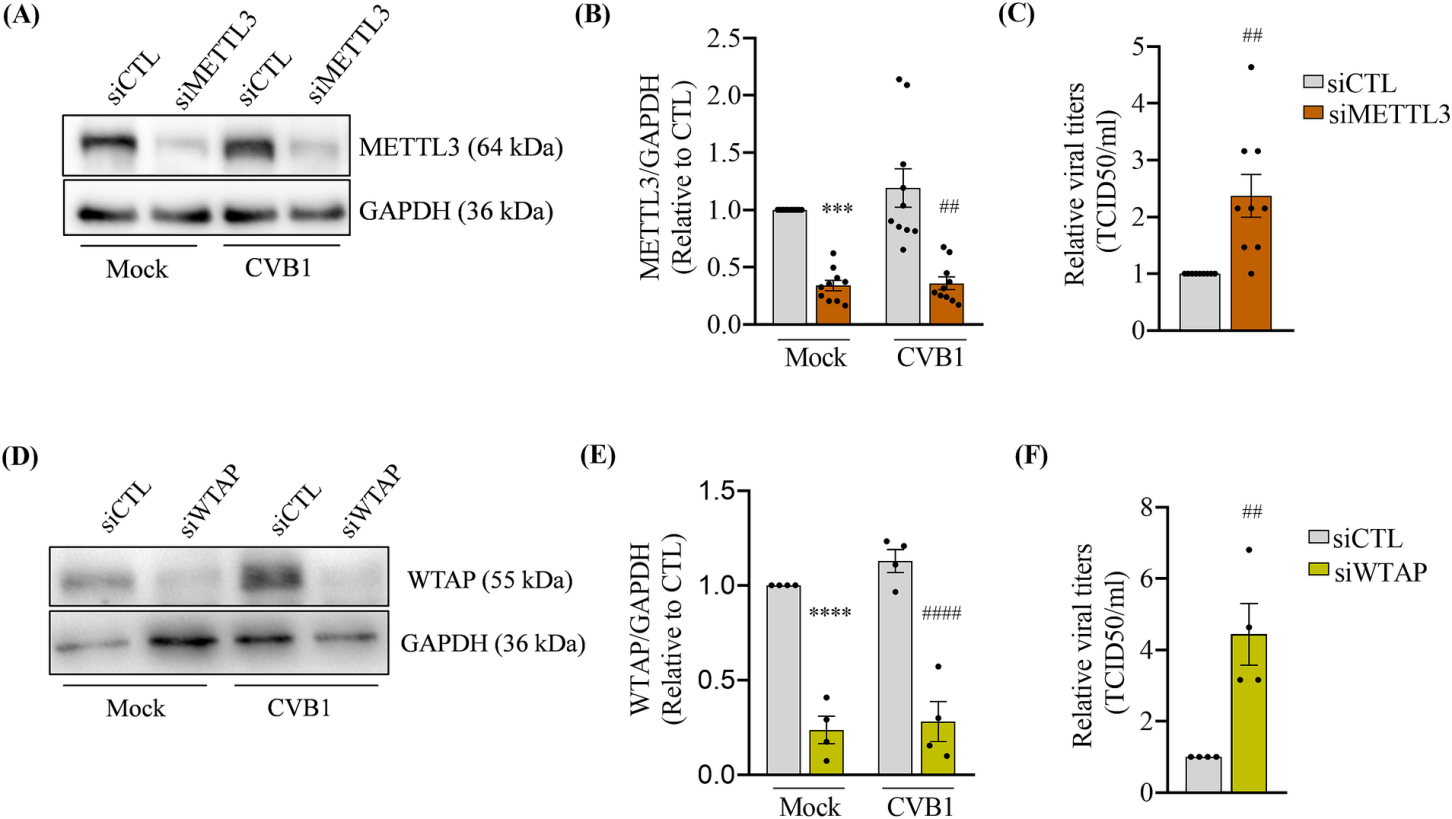
The downregulation of m6A writers increases CVB1 amplification in human pancreatic beta cells. EndoC-*β*H1 cells were transfected with one control siRNA (siCTL) or by the combination of 3 different specific siRNAs targeting METTL3 **(A–C)** (*n* = 10) or one siRNA targeting WTAP **(D–F)** (*n* = 4) and infected with CVB1 at MOI 0.01. Protein expression was quantified by Western blot and normalized by GAPDH **(A,B,D,E)** and viral titers were measured by limit dilution assay (TCID50/ml) **(C,E)** 24 h post-infection. ***p < 0.0005, ****p < 0.0001 vs. siCTL mock; ##*p* < 0.005, ####*p* < 0.0001 vs. siCTL CVB1. One-way ANOVA **(B,E)**, unpaired *t*-test **(C,F)**. Results are expressed as mean ± SEM of 4–10 experiments.

After silencing METTL3 or transfection with control siRNA (Figures 1A,B), EndoC-βH1 cells were infected with CVB1 and collected 24 h post-infection. The analysis of viral titer in the cellular supernatant 24 h post infection indicated that reduction of METTL3 expression led to an increase in CVB1 production (Figure 1C). Consistently, the silencing of Wilms Tumor 1-Associating Protein (WTAP), a key protein regulating the methyltransferase complex (Ping et al., 2014) also led to an increase in CVB1 viral titers when compared to the control condition (Figures 1D–F). These results suggest that decreasing the activity of the methyltransferase complex responsible for the writing of m6A favors CVB1 amplification in EndoC-βH1 cells.

### 3.2 The m6A erasers FTO and ALKBH5 favor CVB1 amplification in EndoC-βH1 cells

**Figure 2:**
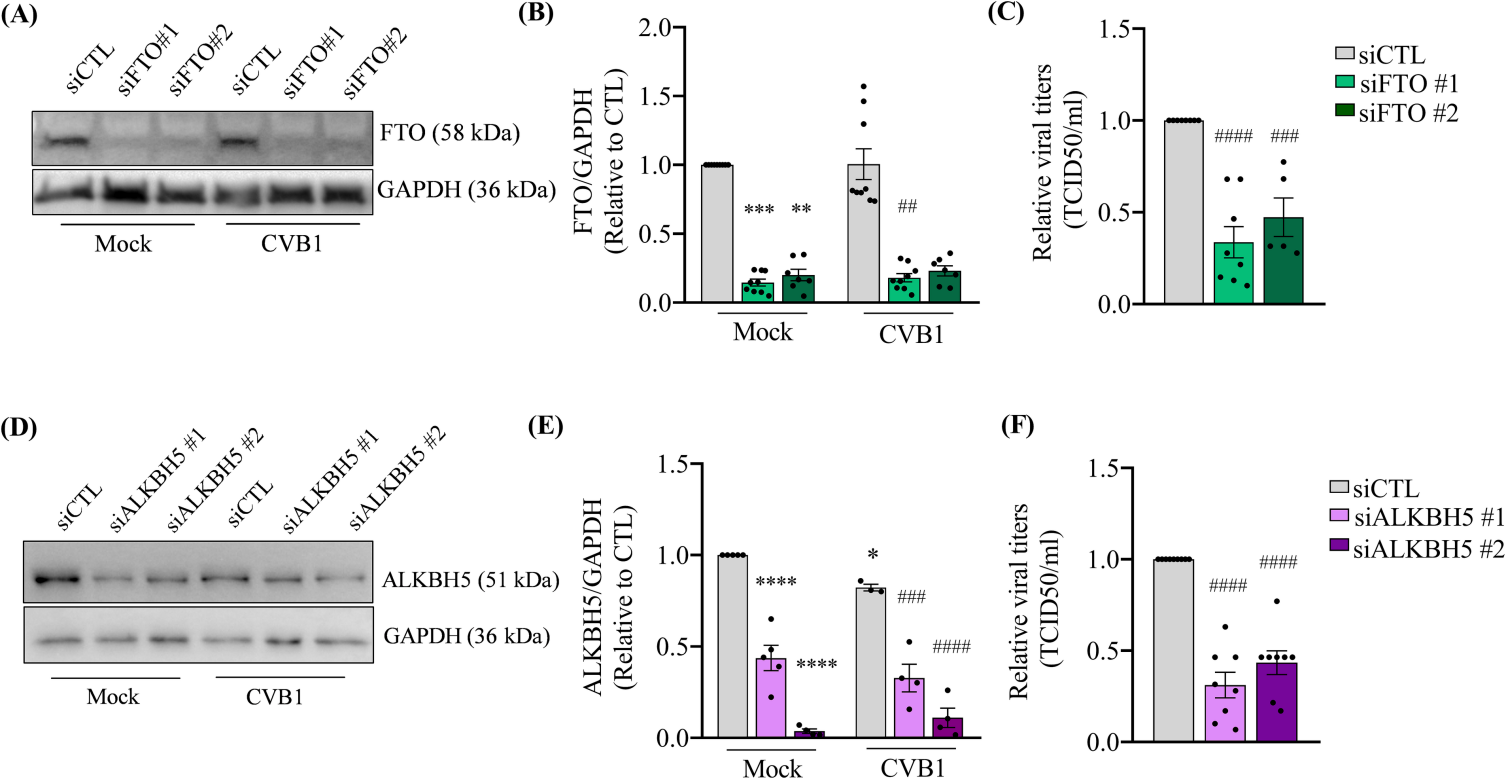
The downregulation of m6A erasers reduces CVB1 amplification in human pancreatic beta cells. EndoC-βH1 cells were transfected with one control siRNA (siCTL) or by 2 different specific siRNAs targeting FTO **(A–C)** (*n* = 5–9) or ALKBH5 **(D–F)** (*n* = 4–9) and infected with CVB1 at M.O.I. 0.01 48 h post-transfection. Protein expression was quantified by Western blot and normalized by GAPDH **(A,B,D,E)** and viral titers were measured by limit dilution assay (TCID50/ml) **(C,F)** 24 h post-infection. *p ≤ 0.05; **p < 0.005, ***p < 0.0005, ****p < 0.0001 vs. siCTL mock; ##*p* < 0.005, ###*p* < 0.0005, ####*p* < 0.0001 vs. siCTL CVB1. One-way ANOVA. Results are expressed as mean ± SEM of 4–9 experiments.

On the contrary the silencing of the demethylases responsible for the elimination of the m6A modification on RNA, namely the Fat Mass And Obesity-Associated Protein (FTO) (Figures 2A,B) and the AlkB Homolog 5, RNA Demethylase (ALKBH5) (Figures 2D,E), leads to a decrease in the production of CVB1 infectious virus 24 h post infection (Figures 2C,F). We also observed a slight reduction in ALKBH5 protein expression after CVB1 infection (Figures 2D,E). Altogether these results indicate that the m6A machinery impacts the viral cycle of CVB1 in the pancreatic beta cell line EndoC-βH1, indicating that high m6A levels are deleterious to virus amplification.

### 3.3 Interferon response to CVB1 infection remains unchanged by METTL3 silencing

**Figure 3:**
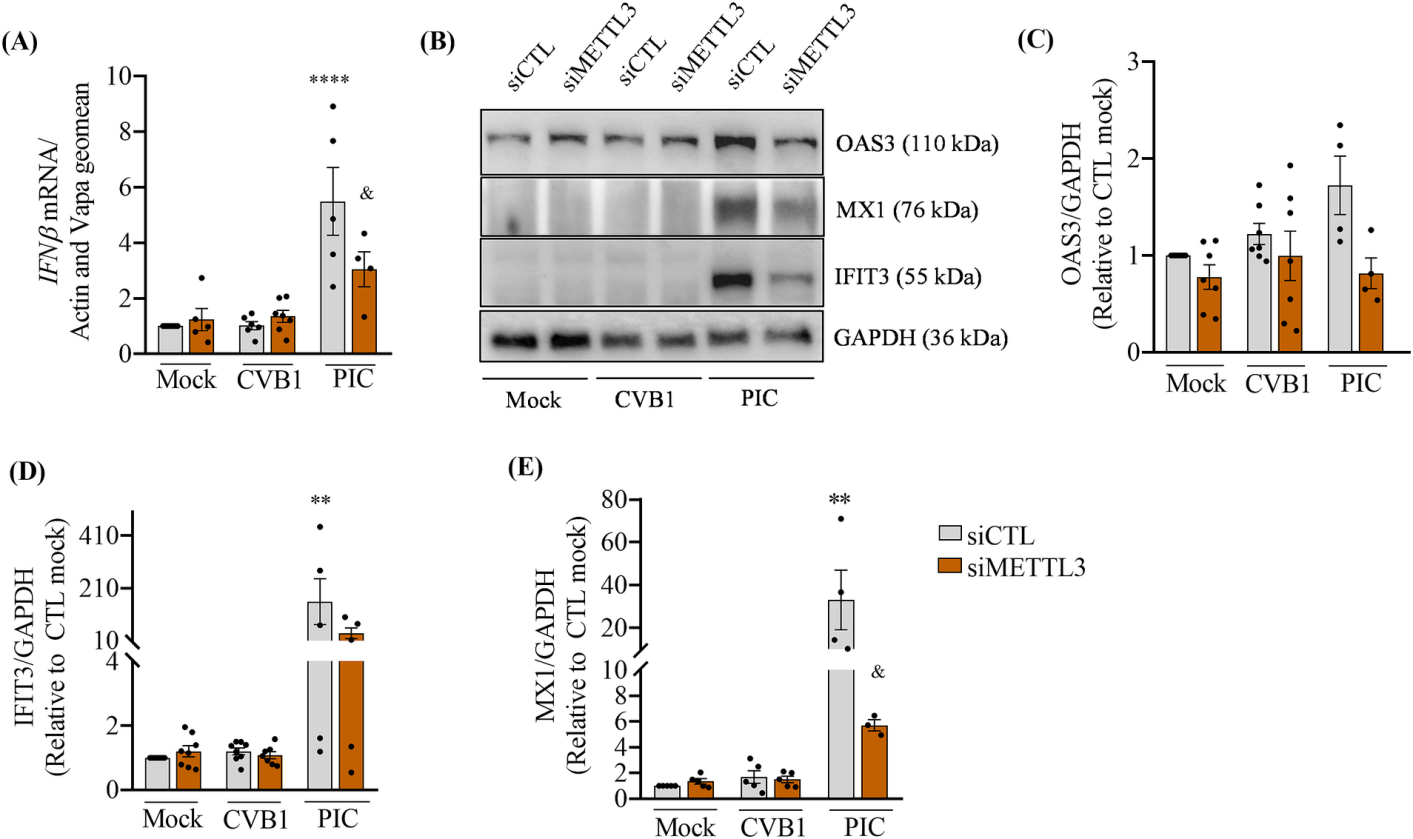
CVB1 infection does not induce a type 1 IFN antiviral response in EndoC-βH1 cells and this is not affected by the silencing of METTL3. EndoC-βH1 cells were transfected with a control siRNA (siCTL) or by the combination of 3 different specific siRNAs targeting METTL3. After 96 h of recovery, cells were transfected with 1 μg/mL of Poly-IC (PIC) for 8 h or infected by CVB1 at M.O.I. 0.01. The cells were collected 24 h post-transfection or infection, the expression of IFNβ mRNA **(A)** was analyzed by RT-qPCR and the values were normalized by the geometric mean of β-actin and VAPA; the protein expression of the ISGs OAS3, MX1 and IFIT3 **(B–E)** were measured by Western blot and normalized by GAPDH. **p < 0.005, ****p < 0.0001 vs. siCTL mock, ^&^*p* < 0.05 vs. siCTL PIC. One-way ANOVA. Results are expressed as mean ± SEM of 4–7 experiments.

METTL3 has been described to affect the cellular antiviral response associated to the production of type 1 interferon (IFN) and to the response to type 1 IFN treatment (Karandashov et al., 2024; De Jesus et al., 2024; McFadden et al., 2021; Winkler et al., 2019). METTL3-mediated m6A modification amplifies the type I IFN responses during infection by VSV or HSV-1 by increasing the translation of IFN-stimulated genes (Chen et al., 2022). We next investigated whether the silencing of METTL3 favors CVB1 amplification by decreasing the CVB1 induced-antiviral response. Cells treated with siRNAs specific for METTL3 or control siRNA were transfected by polyIC (PIC), a mimic of double stranded RNA, or infected by CVB1. The type I interferon IFN*β* gene was induced in PIC transfected cells 24 h after treatment, while no induction of IFNβ was detected in CVB1 infected cells as compared to the to mock-treated ones (Figure 3A). In line with this, the expression of the type 1 IFN-induced proteins OAS3, MX1 and IFIT3 was increased after PIC transfection but not after CVB1 infection (Figure 3B–E). As described by Chen and collaborators, the silencing of METTL3 reduces the PIC induced expression of IFNβ, and ISG encoded proteins OAS3 (Figures 3B,C), MX1 (Figures 3B,E), and IFIT3 (Figures 3B,D). In CVB1 infected cells, the silencing of METTL3 did not modify the absence of CVB1-induced antiviral response. The lack of a type 1 IFN response in EndoC-βH1 cells following CVB1 infection, regardless of METTL3 expression levels, suggests that the regulation of CVB1 replication by the m6A methylation machinery is independent of the cellular antiviral response and is not linked to interferon-mediated defense mechanisms.

### 3.4 The inhibition of FTO blocks CVB1 amplification in EndoC-βH1 cells and in iPSC-derived islet-like cells

**Figure 4:**
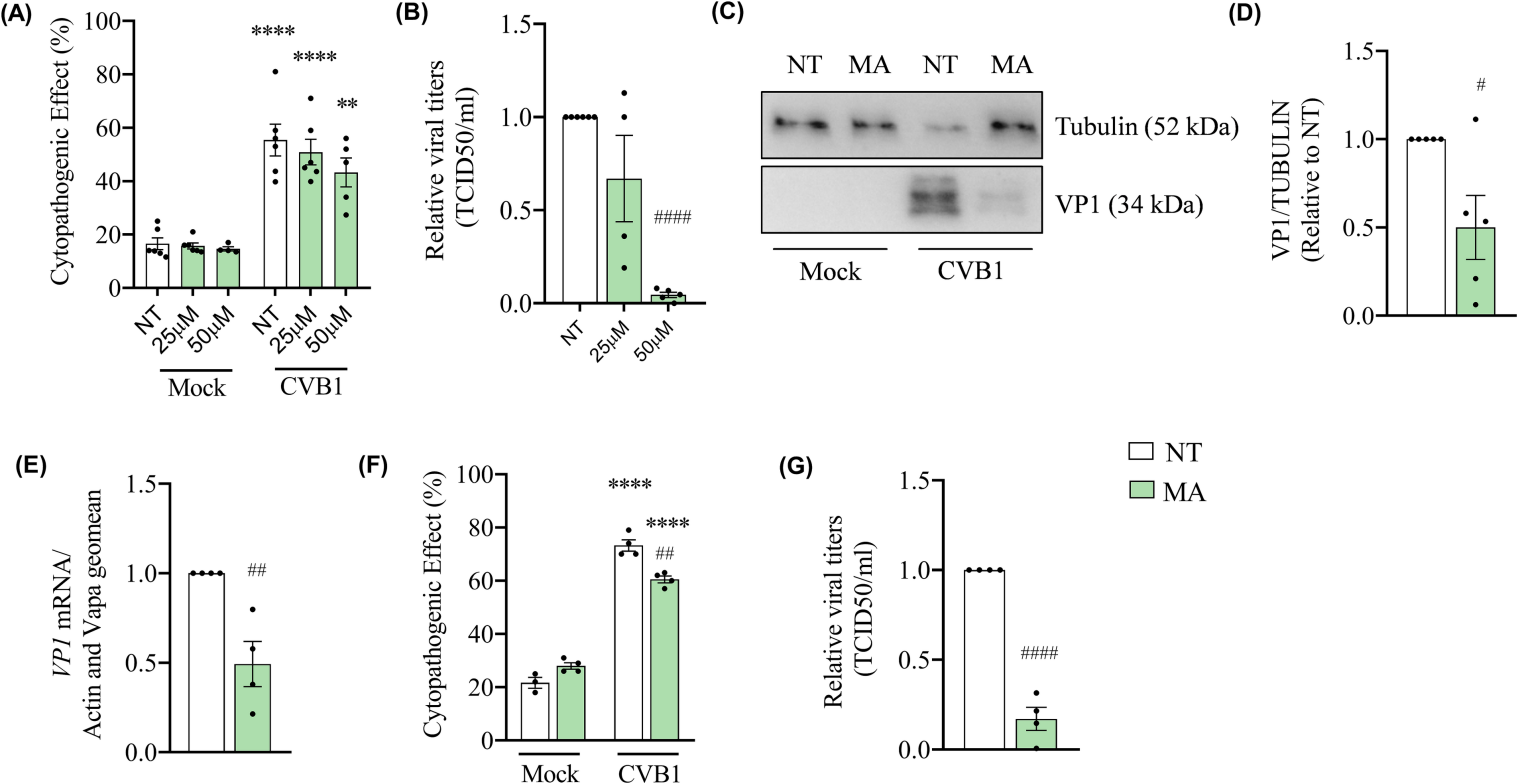
The inhibition of FTO by meclofenamic acid (MA) strongly reduces CVB1 production and viral translation in human pancreatic beta cells. EndoC-βH1 cells were treated with MA 25 μM **(A,B)** or 50 μM **(A–E)** and iPSC—derived islets were treated with MA (25 μM) **(F,G)** or mock treated (NT) at the time of infection by CVB1 at M.O.I. 0.01 **(A–E)** (*n* = 4–6) or M.O.I. 0.05 **(F,G)** (*n* = 3–4). At 24 h post infection, the cytopathogenic effect was quantified by direct cell counting after Hoechst 33342 + propidium iodide staining **(A,F)**, viral titers was measured by end-point dilution assay (TCID50/ml) **(B,G)**, the expression of VP1 viral protein was measured by Western blot **(C,D)** and the expression of VP1 mRNA was analyzed by RT-qPCR and the values were normalized by the geometric mean of β-actin and VAPA **(E)**. **p < 0.005, ****p < 0.0001 vs. NT mock; #*p <* 0.05, ##*p* < 0.005, ####*p* < 0.0001 vs. NT CVB1. One-way ANOVA **(A,B,F)**, unpaired *t*-test **(D,E,G)**. Results are expressed as mean ± SEM of 3–6 experiments.

The gene silencing approach in β-cells based on siRNAs usually requires several days before a significant decrease of the targeted protein is observed (Moore et al., 2012). In EndoC-βH1 cells the silencing of target genes was initiated 3 days before the infection, the impact of the silencing can be both due to a direct effect of the m6A machinery on the viral genome or an indirect effect on the expression of key cellular genes involved in the virus amplification. To determine if the m6A machinery directly affects CVB1 genome, we treated cells with the inhibitor of FTO meclofenamic acid (MA) simultaneously with CVB1 infection. Dose response experiments were performed at the concentrations of 25 μM and 50 μM to evaluate the impact of the treatment on cell viability and CVB1 amplification (Figures 4A,B). At the sublethal concentration of 50 μM (Figure 4A), MA reduced the production of infectious virus by 95% (Figure 4B), the viral capsid protein (VP1) expression (Figures 4C,D) and the replication of the viral genome by 50% (Figure 4E), at 24 h post infection. To test whether the inhibitory effect of MA on CVB1 can be detected in another human pancreatic islet cell model, dispersed induced pluripotent stem cell (iPSC)-derived islet-like were treated or not with MA during infection with CVB1 or mock infection. Since MA at 50 μM impairs the viability of iPSC cells, the concentration of 25 μM was used in these cells (Supplementary Figure S3). In this model, the treatment with MA at sublethal concentration (Figure 4F) also reduced by 75% the production of CVB1 infectious virus in the supernatant of infected culture (Figure 4G) when compared to the non-treated condition. This observation indicates that MA impairs CVB1 production in both EndoC-βH1 human beta cells line and in human iPSC-derived islet-like cells.

### 3.5 The inhibition of FTO rapidly impairs CVB1 replication and translation

**Figure 5:**
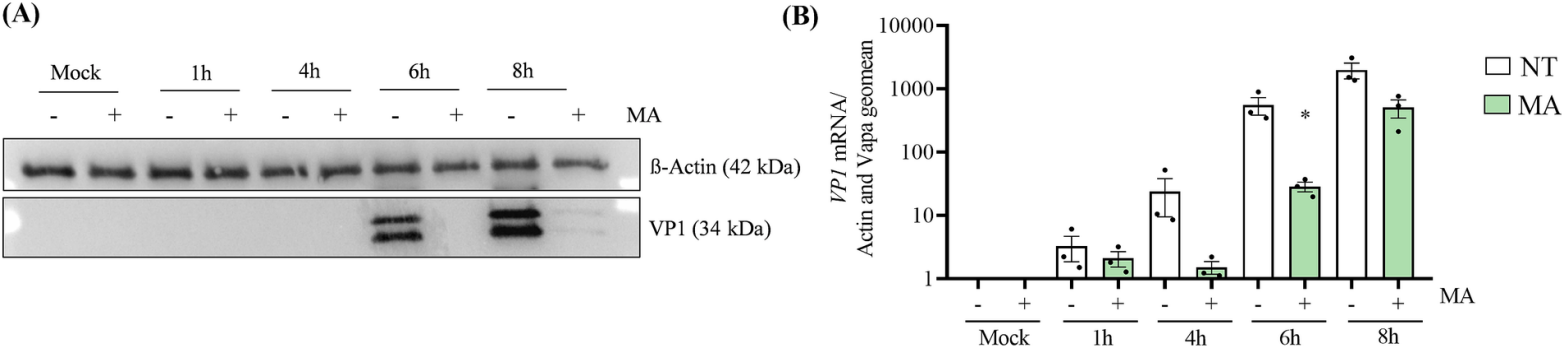
The inhibition of FTO affects CVB1 translation and replication in early infection. EndoC-βH1 cells were treated with MA (50 μM) or mock treated (NT) at the time of infection by CVB1 M.O.I. 1 (*n* = 3). At 1 h, 4 h, 6 h, and 8 h post infection, the expression of VP1 viral protein was measured by Western blot **(A)** and the expression of VP1 mRNA was analyzed by RT-qPCR and the values were normalized by the geometric mean of β-actin and VAPA **(B)**. **(A)** Shows a representative image of 3 independent experiments. *p < 0.05 vs. NT mock, unpaired *t*-test. Results are expressed as mean ± SEM of 3 experiments.

M6A has been described to impact the replication and translation of mRNA and of viral RNA genome. To study the importance of m6A in CVB1 translation and replication we performed time course experiments at early time point post infection. At M.O.I. 1 the VP1 protein is detected at 6 h post infection but not in the presence of MA (Figure 5A). Consistently, at 4 h and 6 h post infection, CVB1 genome quantification is lower in the presence of MA (Figure 5B). Altogether our data show that inhibiting FTO rapidly interferes with CVB1 replication and expression. This observation suggests that FTO favors at least partly CVB1 amplification through a direct effect on the viral genome.

**Figure 6:**
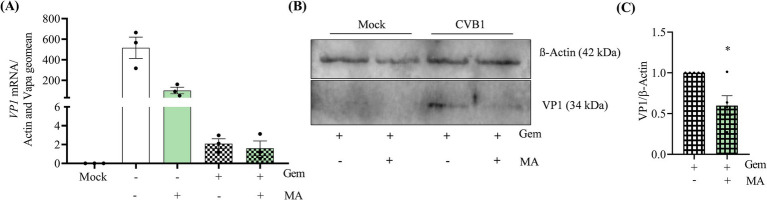
The inhibition of FTO decreases the translation of the incoming CVB1 genome in human pancreatic beta cells. EndoC-βH1 cells were treated with MA (50 μM) in combination or not with Gemcitabine (2 μM) at the time of infection by CVB1 at M.O.I. 10. At 6 h post infection, the expression of VP1 mRNA was analyzed by RT-qPCR and the values were normalized by the geometric mean of β-actin and VAPA **(A)** and the expression of VP1 viral protein was measured by western and normalized by β-actin **(B,C)**. *p < 0.05, unpaired *t*-test. Results are expressed as mean ± SEM of 3–5 experiments.

CVB1 is translated by a cap-independent mechanism driven by an internal ribosome entry site (IRES) at 5′ untranslated region (5’UTR) of the viral genome. Viral translation leads to the production of a long polyprotein which is processed in 11 mature proteins including the viral RNA polymerase 3D. Thus, CVB1 replication and translation are interdependent. To identify whether CVB1 translation is affected by m6A modifications, we investigated the impact of MA on protein expression in the absence of replication using the viral polymerase inhibitor Gemcitabine (Song et al., 2017). The concentration of Gemcitabine to reach a complete inhibition of CVB1 replication was set up in dose response experiments to evaluate the toxicity and the efficacy of the drug (Supplementary Figure S4). The concentration of 2 μM of Gemcitabine allows a 4-log reduction of viral replication (Supplementary Figure S4B) with low toxicity (Supplementary Figure S4A) and complete inhibition of CVB1 production (Supplementary Figure S4C). To analyze the impact of MA on CVB1 translation, we set up a condition of infection allowing the detection of viral protein expression in the absence of replication. The expression of VP1 was detected 6 h post-infection at M.O.I. 1, 10, and 100, but only M.O.I. 10 and 100 allowed the detection of VP1 in the presence of 2 μM Gemcitabine (Supplementary Figure S4D). At 6 h post infection at M.O.I. 10, the amplification of CVB1 genome is reduced by 99% in the presence of 2 μM Gemcitabine (Supplementary Figure S4E; Figure 6A). In the absence of CVB1 replication, MA reduces by 50% the expression of VP1 in CVB1 infected EndoC-βH1 cells (Figures 6B,C). Noteworthy, the number of copies of the viral RNA 6 h post infection was similar when comparing the control condition against treatments with the viral polymerase inhibitor Gemcitabine or the FTO inhibitor MA (Figure 6A). This suggests that the stability of the RNA viral genome is not affected by the MA treatment. Altogether, these data indicate that FTO inhibition hampers CVB1 amplification by acting at the early steps of the viral cycle at least in part by blocking or delaying the translation of the viral genome.

## 4 Discussion

We presently show that the modulation of the expression or activity of the cellular enzymes responsible for the methylation of RNA adenosine (m6A) impacts the amplification of CVB1 in human pancreatic beta cells. The observation that the silencing of m6A writer increases CVB1 amplification while the silencing of erasers decreases CVB1 amplification, suggests that the abundance of m6A is deleterious to CVB1 viral cycle. The ultimate mechanisms by which m6A modulates CVB1 amplification remains to be clarified. Time course experiments indicate that CVB1 translation and replication are delayed in the presence of FTO inhibitor within hours post infection, indicating a direct effect of FTO on the RNA viral genome.

The role of m6A in the modulation of single-stranded positive RNA viruses, whose viral cycle is strictly cytoplasmic, such as CVB, has been documented (Brocard et al., 2017). However, the exact mechanisms behind the addition of m6A in cytoplasmic viruses remain incompletely understood. In this study, we demonstrate that both METTL3 and WTAP negatively regulate the production of CVB1 in pancreatic beta cells. This aligns with the findings of Sacco et al., who revealed that WTAP is essential for directing the METTL3/METTL14 methyltransferase complex to HCV RNA. This recruitment plays a pivotal role in negatively regulating HCV replication and viral particle production (Sacco et al., 2022). Notably, the inhibitory effect of WTAP and METTL3 is reversed upon their knockdown, which leads to enhanced viral replication, similarly to what we observed in cells infected by CVB1.

We cannot exclude an off-target effect on CVB1 amplification when silencing METTL3 or WTAP using siRNAs, which is a potential limitation for the interpretation of our data. Nevertheless, independent silencing of two proteins of the methyltransferase complex, WTAP and METTL3, led to a similar increase of CVB1 amplification, suggesting that m6A methyltransferase activity is deleterious for CVB1 amplification. Moreover, the silencing, by 2 independent siRNAs of the m6A demethylases FTO or ALKBH5, and the inhibition of FTO by MA, all led to a decrease of CVB1 production. Altogether, these data suggest that the presence of m6A counteracts CVB1 viral cycle in EndoC-βH1 cells.

The presence of m6A modification leads to the recruitment of proteins called readers that directly bind to these modified residues. The consequence of this binding can be pro-viral or anti-viral depending on the position of the modification. For instance, the binding of the m6A reader YTHDF2 to the viral genome of HCV in the region coding for E1 leads to a decreased packaging into newly produced virions in the cytoplasm of infected cells (Gokhale et al., 2016) and is thus antiviral. On the contrary, the binding of the reader YTHDF2 to m6A modified motifs in 3’-UTR of HCV RNA inhibits the recognition of viral RNA by retinoic acid-inducible gene-I (RIG-I), leading to the immune evasion of HCV (Ge et al., 2023) and is thus pro-viral. Thus, the impact of m6A modifications in the viral genome depends on the site of the modification and on the readers involved. In enterovirus 71 (EV71), a member of the same enterovirus family as CVB, METTL3 interacts with the viral RNA-dependent RNA polymerase 3D, enhancing its SUMOylation and ubiquitination, which in turn promotes viral replication (Hao et al., 2019) or translation (Yao et al., 2020). Consistently, silencing METTL3 reduces EV71 amplification. Similarly, the silencing of METTL3 or its cofactor METTL14 reduces the amplification of CVB3 in HeLa cells, and silencing of FTO and ALKBH5 increases virus amplification (Zhao H. et al., 2024). In contrast, we presently observed that METTL3 silencing in EndoC-βH1 cells leads to increased production of CVB1, highlighting distinct regulatory mechanisms for these related viruses. This may result from differences in the m6A modification profiles of these enteroviruses or from cell-specific recruitment of m6A reader proteins. Indeed, during EV71 infection, depletion of m6A readers like YTHDF2 and YTHDF3 in Vero cells reduced viral replication; however, this same depletion in RD cells had a pro-viral effect, further illustrating variations in the role of m6A readers across different cell lines (Hao et al., 2019). Additionally, strain-specific variations in m6A profiles were observed in HIV-1, with mapping of m6A sites on the HIV-1BaL and HIV-1JR-CSF strains revealing additional YTHDF reader protein binding sites compared to the NL4-3 strain, suggesting that viral genomic differences influence m6A deposition and reader protein interactions (Phillips et al., 2024). The end result of the inhibition or silencing of the m6A machinery players will reflects the combined pro- and anti-viral effects of these modifications, both in the viral genome and the cellular transcriptome. Our data suggest that a high level of m6A impairs CVB1 translation, but identification of the readers and sites involved need further study.

The m6A-regulation in IRES-dependent translation virus, such as CVB1, has been described for HCV (Kim and Siddiqui, 2021). HCV translation requires the recruitment of the YTHDF2 reader in the IRES and depends on the RNA helicase activity of YTHDF2. The translation of CVB1 is modulated by the inhibition of FTO, the potential role of the YTHDF2 helicase or the translational factor eIF3 (Fukaya et al., 2016), another m6A reader, needs further investigation. Interestingly, the inhibition of FTO leads to a delayed expression and replication of the viral genome at early time points but drastically impairs the production of infectious virus at 24 h post infection. This observation supports the hypothesis that later steps of the viral cycle such as assembly, release or maturation of the virion, are also affected by m6A modification. The impact of the m6A machinery on these late steps of the viral cycle deserve further investigation. The clear identification of the methylated residues in CVB1 genome, the readers bound to these m6A modified positions, and the impact of this binding on each step of the viral cycle will help to further detail the interactions between CVB1 and the human pancreatic b-cells.

The role of m6A in the regulation of antiviral innate immunity has been extensively studied (Karandashov et al., 2024; Aufgebauer et al., 2024). M6A regulates the expression of type 1 IFN genes and a series of IFN-stimulated genes, including also the Rig-Like Receptor (RLR) and Toll-Like Receptor sensing pathways, key sensors of virus infection. In particular, the m6A modification on viral RNA can prevent its recognition by the pattern recognition receptors RIG-1 and MDA5, and consequently interfere with the innate antiviral response leading to an increased viral amplification (Aufgebauer et al., 2024; Kim et al., 2020; Lu et al., 2020; Qiu et al., 2021). In EndoC-βH1 cells, we observed that the silencing of METTL3 is associated with an increase in CVB1 amplification, but no innate antiviral response is detected in CVB1 infected cells, regardless of METTL3 expression level. This observation suggests that the m6A does not affect CVB1 amplification by modulating the detection of the viral genome by RLR in EndoC-βH1.The absence of innate antiviral response in CVB infected rat pancreatic beta cells has already been described (Marroqui et al., 2015), while pancreatic alpha cells are capable to mount an efficient and virus-controlling antiviral response (Marroqui et al., 2015; Eizirik et al., 2023). The control of the innate antiviral response has been described previously for enterovirus and relies on the activity of 2 key viral proteases 2A^pro^ and 3C^pro^, on the non-structural protein 2C and on the RNA dependent RNA polymerase 3D^pol^ (Wei et al., 2024; Zhao X. et al., 2024). According to our data, the cellular mechanisms at work in the control of the antiviral response in CVB1 infected EndoC-*β*H1 cells are not affected by the modulation of the m6A machinery. The CVB1 ATCC strain Conn-5, described in the present paper, induces interferon response in PBMC cells and in pancreatic ductal cells PANC-1 (Hamalainen et al., 2014; Buchacher et al., 2022). In these cells, the m6A machinery could impact the CVB1 amplification directly, by acting on the viral genome, and/or indirectly by modulating the CVB1-induced antiviral response. In the context of T1D, where type I IFNα is detected in pancreatic islets (Huang et al., 1995; Richardson et al., 2014), there is both METTL3 induction and hypermethylation of antiviral genes (De Jesus et al., 2024). On the other hand, the absence of a clear antiviral response in CVB1 infected EndoC-βH1cells (present data), allowed us to observe a direct effect of the m6A machinery on CVB1 amplification without the context of an antiviral response.

Enterovirus expression is detected in the pancreas of diabetic donors and newly diagnosed living patients (Richardson et al., 2009; Apaolaza et al., 2021; Krogvold et al., 2015). These enteroviruses, recovered from pancreatic biopsies, are infectious but poorly cytopathogenic and replicative (Oikarinen et al., 2021; Krogvold et al., 2022). The molecular mechanisms behind this persistence and reduced replication remain unclear. M6A methylation is dynamically regulated during T1D progression, with METTL3 levels peaking at disease onset and subsequently declining (De Jesus et al., 2024). This regulation may impact the innate immune response by balancing the expression and stability of immune mediators, thus regulating enteroviral infections. Together, these findings raise the hypothesis that m6A dynamics could play a role in both antiviral responses and enterovirus persistence in pancreatic *β*-cells, contributing to the pathogenesis of T1D.

## Data Availability

The raw data supporting the conclusions of this article will be made available by the authors, without undue reservation.

## References

[ref1] Alvelos M. I. Szymczak F. Castela A. Marin-Canas S. De Souza B. M. Gkantounas I.. (2021). A functional genomic approach to identify reference genes for human pancreatic beta cell real-time quantitative RT-PCR analysis. Islets 13, 51–65. doi: 10.1080/19382014.2021.1948282 34241569 PMC8280887

[ref2] Apaolaza P. S. Balcacean D. Zapardiel-Gonzalo J. Nelson G. Lenchik N. Akhbari P.. (2021). Islet expression of type I interferon response sensors is associated with immune infiltration and viral infection in type 1 diabetes. Sci. Adv. 7:eabd6527. doi: 10.1126/sciadv.abd6527, PMID: 33627420 PMC7904254

[ref3] Aufgebauer C. J. Bland K. M. Horner S. M. (2024). Modifying the antiviral innate immune response by selective writing, erasing, and reading of m(6)a on viral and cellular RNA. Cell Chem. Biol. 31, 100–109. doi: 10.1016/j.chembiol.2023.12.004, PMID: 38176419 PMC10872403

[ref4] Boccaletto P. Machnicka M. A. Purta E. Piatkowski P. Baginski B. Wirecki T. K.. (2018). Modomics: a database of Rna modification pathways. 2017 update. Nucleic Acids Res. 46, D303–d307. doi: 10.1093/nar/gkx1030, PMID: 29106616 PMC5753262

[ref5] Brocard M. Ruggieri A. Locker N. (2017). m6A Rna methylation, a new hallmark in virus-host interactions. J. Gen. Virol. 98, 2207–2214. doi: 10.1099/jgv.0.000910, PMID: 28869001

[ref6] Buchacher T. Honkimaa A. Valikangas T. Lietzen N. Hirvonen M. K. Laiho J. E.. (2022). Persistent coxsackievirus B1 infection triggers extensive changes in the transcriptome of human pancreatic ductal cells. iScience 25:103653. doi: 10.1016/j.isci.2021.103653, PMID: 35024587 PMC8728469

[ref7] Chen J. Wei X. Wang X. Liu T. Zhao Y. Chen L.. (2022). Tbk1-Mettl3 axis facilitates antiviral immunity. Cell Rep. 38:110373. doi: 10.1016/j.celrep.2022.110373, PMID: 35172162

[ref8] Cosentino C. Toivonen S. Diaz Villamil E. Atta M. Ravanat J.-L. Demine S.. (2018). Pancreatic β-cell trna hypomethylation and fragmentation link Trmt10A deficiency with diabetes. Nucleic Acids Res. 46, 10302–10318. doi: 10.1093/nar/gky839, PMID: 30247717 PMC6212784

[ref9] De Jesus D. F. Zhang Z. Brown N. K. Li X. Xiao L. Hu J.. (2024). Redox regulation of m(6)a methyltransferase Mettl3 in β-cells controls the innate immune response in type 1 diabetes. Nat. Cell Biol. 26, 421–437. doi: 10.1038/s41556-024-01368-0, PMID: 38409327 PMC11042681

[ref10] Eizirik D. L. Szymczak F. Mallone R. (2023). Why does the immune system destroy pancreatic beta-cells but not alpha-cells in type 1 diabetes? Nat. Rev. Endocrinol. 19, 425–434. doi: 10.1038/s41574-023-00826-3 37072614

[ref11] Fantuzzi F. Toivonen S. Schiavo A. A. Chae H. Tariq M. Sawatani T.. (2022). In depth functional characterization of human induced pluripotent stem cell-derived beta cells *in vitro* and *in vivo*. Front. Cell Dev. Biol. 10:967765. doi: 10.3389/fcell.2022.967765 36060810 PMC9428245

[ref12] Fukaya M. Brorsson C. A. Meyerovich K. Catrysse L. Delaroche D. Vanzela E. C.. (2016). A20 inhibits beta-cell apoptosis by multiple mechanisms and predicts residual beta-cell function in type 1 diabetes. Mol. Endocrinol. 30, 48–61. doi: 10.1210/me.2015-1176, PMID: 26652732 PMC5414657

[ref13] Ge Y. Tang S. Xia T. Shi C. (2023). Research progress on the role of Rna N(6)-methyladenosine methylation in Hcv infection. Virology 582, 35–42. doi: 10.1016/j.virol.2023.03.007 36996690

[ref14] Gokhale N. S. Mcintyre A. B. R. Mcfadden M. J. Roder A. E. Kennedy E. M. Gandara J. A.. (2016). N6-Methyladenosine in Flaviviridae viral Rna genomes regulates infection. Cell Host Microbe 20, 654–665. doi: 10.1016/j.chom.2016.09.015, PMID: 27773535 PMC5123813

[ref15] Gregory G. A. Robinson T. I. G. Linklater S. E. Wang F. Colagiuri S. De Beaufort C.. (2022). Global incidence, prevalence, and mortality of type 1 diabetes in 2021 with projection to 2040: a modelling study. Lancet Diabetes Endocrinol. 10, 741–760. doi: 10.1016/S2213-8587(22)00218-2, PMID: 36113507

[ref16] Hamalainen S. Nurminen N. Ahlfors H. Oikarinen S. Sioofy-Khojine A. B. Frisk G.. (2014). Coxsackievirus B1 reveals strain specific differences in plasmacytoid dendritic cell mediated immunogenicity. J. Med. Virol. 86, 1412–1420. doi: 10.1002/jmv.23903, PMID: 24616040

[ref17] Hao H. Hao S. Chen H. Chen Z. Zhang Y. Wang J.. (2019). N6-methyladenosine modification and Mettl3 modulate enterovirus 71 replication. Nucleic Acids Res. 47, 362–374. doi: 10.1093/nar/gky1007, PMID: 30364964 PMC6326802

[ref18] Honkanen H. Oikarinen S. Nurminen N. Laitinen O. H. Huhtala H. Lehtonen J.. (2017). Detection of enteroviruses in stools precedes islet autoimmunity by several months: possible evidence for slowly operating mechanisms in virus-induced autoimmunity. Diabetologia 60, 424–431. doi: 10.1007/s00125-016-4177-z, PMID: 28070615

[ref19] Horner S. M. Reaves J. V. (2024). Recent insights into N(6)-methyladenosine during viral infection. Curr. Opin. Genet. Dev. 87:102213. doi: 10.1016/j.gde.2024.102213, PMID: 38901100 PMC11317213

[ref20] Huang X. Yuang J. Goddard A. Foulis A. James R. F. Lernmark A.. (1995). Interferon expression in the pancreases of patients with type I diabetes. Diabetes 44, 658–664. doi: 10.2337/diab.44.6.658, PMID: 7540571

[ref21] Jiang X. Liu B. Nie Z. Duan L. Xiong Q. Jin Z.. (2021). The role of m6A modification in the biological functions and diseases. Signal Transduct. Target. Ther. 6:74. doi: 10.1038/s41392-020-00450-x, PMID: 33611339 PMC7897327

[ref22] Karandashov I. Kachanov A. Dukich M. Ponomareva N. Brezgin S. Lukashev A.. (2024). M(6)a methylation in regulation of antiviral innate immunity. Viruses 16:601. doi: 10.3390/v16040601, PMID: 38675942 PMC11054785

[ref23] Kim G. W. Imam H. Khan M. Siddiqui A. (2020). N(6)-Methyladenosine modification of hepatitis B and C viral Rnas attenuates host innate immunity via rig-I signaling. J. Biol. Chem. 295, 13123–13133. doi: 10.1074/jbc.RA120.014260 32719095 PMC7489896

[ref24] Kim G. W. Siddiqui A. (2021). N6-methyladenosine modification of Hcv Rna genome regulates cap-independent Ires-mediated translation via Ythdc2 recognition. Proc. Natl. Acad. Sci. USA 118:e2022024118. doi: 10.1073/pnas.2022024118 33649237 PMC7958429

[ref25] Kim K. W. Horton J. L. Pang C. N. I. Jain K. Leung P. Isaacs S. R.. (2019). Higher abundance of enterovirus a species in the gut of children with islet autoimmunity. Sci. Rep. 9:1749. doi: 10.1038/s41598-018-38368-8, PMID: 30741981 PMC6370883

[ref26] Krogvold L. Edwin B. Buanes T. Frisk G. Skog O. Anagandula M.. (2015). Detection of a low-grade enteroviral infection in the islets of langerhans of living patients newly diagnosed with type 1 diabetes. Diabetes 64, 1682–1687. doi: 10.2337/db14-1370, PMID: 25422108

[ref27] Krogvold L. Leete P. Mynarek I. M. Russell M. A. Gerling I. C. Lenchik N. I.. (2022). Detection of antiviral tissue responses and increased cell stress in the pancreatic islets of newly diagnosed type 1 diabetes patients: results from the DiViD study. Front. Endocrinol. (Lausanne) 13:881997. doi: 10.3389/fendo.2022.881997, PMID: 35957810 PMC9360491

[ref28] Laitinen O. H. Honkanen H. Pakkanen O. Oikarinen S. Hankaniemi M. M. Huhtala H.. (2014). Coxsackievirus B1 is associated with induction of beta-cell autoimmunity that portends type 1 diabetes. Diabetes 63, 446–455. doi: 10.2337/db13-0619, PMID: 23974921

[ref29] Lennette E. H. Schmidt N. J. (Eds.) (1969). “General principles underlying laboratory diagnosis of viral and rickettsial infections” in Diagnostic procedures for viral and rickettsial infections, 1–63.

[ref30] Liu N. Pan T. (2015). Rna epigenetics. Transl. Res. 165, 28–35. doi: 10.1016/j.trsl.2014.04.003, PMID: 24768686 PMC4190089

[ref31] Lu M. Zhang Z. Xue M. Zhao B. S. Harder O. Li A.. (2020). N(6)-methyladenosine modification enables viral Rna to escape recognition by Rna sensor rig-I. Nat. Microbiol. 5, 584–598. doi: 10.1038/s41564-019-0653-9, PMID: 32015498 PMC7137398

[ref32] Lytrivi M. Senée V. Salpea P. Fantuzzi F. Philippi A. Abdulkarim B.. (2021). Dnajc3 deficiency induces β-cell mitochondrial apoptosis and causes syndromic young-onset diabetes. Eur. J. Endocrinol. 184, 455–468. doi: 10.1530/EJE-20-0636, PMID: 33486469

[ref33] Marroqui L. Lopes M. Dos Santos R. S. Grieco F. A. Roivainen M. Richardson S. J.. (2015). Differential cell autonomous responses determine the outcome of coxsackievirus infections in murine pancreatic alpha and beta cells. eLife 4:e06990. doi: 10.7554/eLife.06990, PMID: 26061776 PMC4480275

[ref34] Mcfadden M. J. Mcintyre A. B. R. Mourelatos H. Abell N. S. Gokhale N. S. Ipas H.. (2021). Post-transcriptional regulation of antiviral gene expression by N6-methyladenosine. Cell Rep. 34:108798. doi: 10.1016/j.celrep.2021.108798, PMID: 33657363 PMC7981787

[ref35] Moore F. Cunha D. A. Mulder H. Eizirik D. L. (2012). Use of Rna interference to investigate cytokine signal transduction in pancreatic beta cells. Methods Mol. Biol. 820, 179–194. doi: 10.1007/978-1-61779-439-1_11, PMID: 22131032

[ref36] Nekoua M. P. Mercier A. Alhazmi A. Sane F. Alidjinou E. K. Hober D. (2022). Fighting Enteroviral infections to prevent type 1 diabetes. Microorganisms 10:768. doi: 10.3390/microorganisms10040768, PMID: 35456818 PMC9031364

[ref37] Norris J. M. Johnson R. K. Stene L. C. (2020). Type 1 diabetes—early life origins and changing epidemiology. Lancet Diab. Endocrinol. 8, 226–238. doi: 10.1016/S2213-8587(19)30412-7, PMID: 31999944 PMC7332108

[ref38] Oikarinen M. Tauriainen S. Oikarinen S. Honkanen T. Collin P. Rantala I.. (2012). Type 1 diabetes is associated with enterovirus infection in gut mucosa. Diabetes 61, 687–691. doi: 10.2337/db11-1157, PMID: 22315304 PMC3282798

[ref39] Oikarinen S. Krogvold L. Edwin B. Buanes T. Korsgren O. Laiho J. E.. (2021). Characterisation of enterovirus Rna detected in the pancreas and other specimens of live patients with newly diagnosed type 1 diabetes in the DiViD study. Diabetologia 64, 2491–2501. doi: 10.1007/s00125-021-05525-0, PMID: 34390364 PMC8494699

[ref40] Overbergh L. Valckx D. Waer M. Mathieu C. (1999). Quantification of murine cytokine mrnas using real time quantitative reverse transcriptase Pcr. Cytokine 11, 305–312. doi: 10.1006/cyto.1998.0426 10328870

[ref41] Patterson C. C. Harjutsalo V. Rosenbauer J. Neu A. Cinek O. Skrivarhaug T.. (2019). Trends and cyclical variation in the incidence of childhood type 1 diabetes in 26 European centres in the 25 year period 1989-2013: a multicentre prospective registration study. Diabetologia 62, 408–417. doi: 10.1007/s00125-018-4763-3, PMID: 30483858

[ref42] Phillips S. Mishra T. Huang S. Wu L. (2024). Functional impacts of Epitranscriptomic m(6)a modification on Hiv-1 infection. Viruses 16:127. doi: 10.3390/v16010127, PMID: 38257827 PMC10820791

[ref43] Ping X. L. Sun B. F. Wang L. Xiao W. Yang X. Wang W. J.. (2014). Mammalian Wtap is a regulatory subunit of the Rna N6-methyladenosine methyltransferase. Cell Res. 24, 177–189. doi: 10.1038/cr.2014.3, PMID: 24407421 PMC3915904

[ref44] Poh H. X. Mirza A. H. Pickering B. F. Jaffrey S. R. (2022). Alternative splicing of Mettl3 explains apparently Mettl3-independent m6A modifications in mrna. PLoS Biol. 20:e3001683. doi: 10.1371/journal.pbio.3001683, PMID: 35853000 PMC9295969

[ref45] Qiu W. Zhang Q. Zhang R. Lu Y. Wang X. Tian H.. (2021). N(6)-methyladenosine Rna modification suppresses antiviral innate sensing pathways via reshaping double-stranded Rna. Nat. Commun. 12:1582. doi: 10.1038/s41467-021-21904-y, PMID: 33707441 PMC7952553

[ref46] Ravassard P. Hazhouz Y. Pechberty S. Bricout-Neveu E. Armanet M. Czernichow P.. (2011). A genetically engineered human pancreatic beta cell line exhibiting glucose-inducible insulin secretion. J. Clin. Invest. 121, 3589–3597. doi: 10.1172/JCI58447, PMID: 21865645 PMC3163974

[ref47] Richardson S. J. Morgan N. G. Foulis A. K. (2014). Pancreatic pathology in type 1 diabetes mellitus. Endocr. Pathol. 25, 80–92. doi: 10.1007/s12022-014-9297-8 24522639

[ref48] Richardson S. J. Willcox A. Bone A. J. Foulis A. K. Morgan N. G. (2009). The prevalence of enteroviral capsid protein vp1 immunostaining in pancreatic islets in human type 1 diabetes. Diabetologia 52, 1143–1151. doi: 10.1007/s00125-009-1276-0, PMID: 19266182

[ref49] Sacco M. T. Bland K. M. Horner S. M. (2022). Wtap targets the Mettl3 m^6^A-methyltransferase complex to cytoplasmic hepatitis C virus RNA to regulate infection. J. Virol. 96, e00997–e00922. doi: 10.1128/jvi.00997-22 36314819 PMC9683008

[ref50] Santin I. Dos Santos R. S. Eizirik D. L. (2016). Pancreatic Beta cell survival and signaling pathways: effects of type 1 diabetes-associated genetic variants. Methods Mol. Biol. 1433, 21–54. doi: 10.1007/7651_2015_291, PMID: 26936771

[ref51] Sioofy-Khojine A. B. Lehtonen J. Nurminen N. Laitinen O. H. Oikarinen S. Huhtala H.. (2018). Coxsackievirus B1 infections are associated with the initiation of insulin-driven autoimmunity that progresses to type 1 diabetes. Diabetologia 61, 1193–1202. doi: 10.1007/s00125-018-4561-y 29404673

[ref52] Song J. H. Kim S. R. Heo E. Y. Lee J. Y. Kim D. E. Cho S.. (2017). Antiviral activity of gemcitabine against human rhinovirus *in vitro* and *in vivo*. Antivir. Res. 145, 6–13. doi: 10.1016/j.antiviral.2017.07.003, PMID: 28705625

[ref53] Vehik K. Lynch K. F. Wong M. C. Tian X. Ross M. C. Gibbs R. A.. (2019). Prospective virome analyses in young children at increased genetic risk for type 1 diabetes. Nat. Med. 25, 1865–1872. doi: 10.1038/s41591-019-0667-0, PMID: 31792456 PMC6898786

[ref54] Wang X. Lu Z. Gomez A. Hon G. C. Yue Y. Han D.. (2014). N6-methyladenosine-dependent regulation of messenger Rna stability. Nature 505, 117–120. doi: 10.1038/nature12730, PMID: 24284625 PMC3877715

[ref55] Wang X. Zhao B. S. Roundtree I. A. Lu Z. Han D. Ma H.. (2015). N(6)-methyladenosine modulates messenger Rna translation efficiency. Cell 161, 1388–1399. doi: 10.1016/j.cell.2015.05.014, PMID: 26046440 PMC4825696

[ref56] Wei J. Lv L. Wang T. Gu W. Luo Y. Feng H. (2024). Recent Progress in innate immune responses to enterovirus A71 and viral evasion strategies. Int. J. Mol. Sci. 25:5688. doi: 10.3390/ijms25115688 38891876 PMC11172324

[ref57] Winkler R. Gillis E. Lasman L. Safra M. Geula S. Soyris C.. (2019). M(6)a modification controls the innate immune response to infection by targeting type I interferons. Nat. Immunol. 20, 173–182. doi: 10.1038/s41590-018-0275-z, PMID: 30559377

[ref58] Yang D. Zhao G. Zhang H. M. (2023). M(6)a reader proteins: the executive factors in modulating viral replication and host immune response. Front. Cell. Infect. Microbiol. 13:1151069. doi: 10.3389/fcimb.2023.1151069, PMID: 37325513 PMC10266107

[ref9001] Yao M. Dong Y. Wang Y. Liu H. Ma H. Zhang H.. (2020). N(6)-methyladenosine modifications enhance enterovirus 71 ORF translation through METTL3 cytoplasmic distribution. Biochem. Biophys. Res. Commun. 527:297–304. 32446384 10.1016/j.bbrc.2020.04.088

[ref59] Zhao H. Gao Z. Sun J. Qiao H. Zhao Y. Cui Y.. (2024). N6-Methyladenosine positively regulates Coxsackievirus B3 replication. Viruses 16:1448. doi: 10.3390/v16091448, PMID: 39339923 PMC11437462

[ref60] Zhao X. Hu Y. Zhao J. Liu Y. Ma X. Chen H.. (2024). Role of protein post-translational modifications in enterovirus infection. Front. Microbiol. 15:1341599. doi: 10.3389/fmicb.2024.1341599, PMID: 38596371 PMC11002909

[ref61] Zhong X. Zhou Z. Yang G. (2024). The functions of N-methyladenosine (m6A) modification on Hiv-1 mrna. Cell Biochem. Biophys. 82, 561–574. doi: 10.1007/s12013-024-01280-2 38753251

