# Supplementary material for: The N6-methyladenosine RNA epigenetic modification modulates the amplification of coxsackievirus B1 in human pancreatic beta cells
Source: Front Microbiol. 2024 Dec 18;15:1501061. doi: 10.3389/fmicb.2024.1501061 (PMC11688287; doi:10.3389/fmicb.2024.1501061)
Supplement: Supplementary file 1 [file Data_Sheet_1.pdf]

Supplementary Materials

Content:

Abbreviations

Tables

Figures

Abbreviations:

ALKBH5 - AlkB Homolog 5, RNA Demethylase

CVB1 – Coxsackievirus B Serotype 1

FTO - Fat mass and obesity-associated protein

IFNB – Interferon Beta

ISG - IFN-stimulated gene

M6A – N6-methyladenosine

METTL3 – Methyltransferase 3

MOI – Multiplicity of infection

PIC - Polyinosinic-polycytidylic acid

Supplementary Table 1

siRNAs	Sequence (5’-3’)	Reference	Supplier
Allstars Negative Control siRNA	Sequence not provided	1027281	Qiagen
siMETTL3 #1	GAACGGGUAGAUGAAAUUAtt	s32141	Thermo Fisher Scientific
siMETTL3 #2	GAUCCUGAGUUAGAGAAGAtt	s32142	Thermo Fisher Scientific
siMETTL3 #3	GCAGUUCCUGAAUUAGCUAtt	s32143	Thermo Fisher Scientific
siWTAP	AGAUCUUAACUCUAAUGAUtt	s18431	Thermo Fisher Scientific
siFTO #1	CAUCCUCAUUGGUAAUCCAtt	s35510	Thermo Fisher Scientific
siFTO #2	CAUUACCUGCUGAUCAGAAtt	s35511	Thermo Fisher Scientific
siALKBH5 #1	GCCUGUUAGGGCUGAAGAAtt	s29686	Thermo Fisher Scientific
siALKBH5 #2	GGCUCAUCCUUACGUAGUUtt	s29688	Thermo Fisher Scientific

Table 1. List of siRNAs used in the present study.

Supplementary Table 2

Gene	Sequence (5'-3')	Primer	Supplier
ACTB	CTGTACGCCAACACAGTGCT	Forward	Eurogentec
	GCTCAGGAGGAGCAATGATC	Reverse	
IFNβ	GTTGAGAACCTCCTGGCTAATG	Forward	Eurogentec
	GGTAATGCAGAATCCTCCCATAAT	Reverse	
MX1	AGACAGGACCATCGGAATCT	Forward	Eurogentec
	GTAACCCTTCTTCAGGTGGAAC	Reverse	
VAPA	TACCGAAACAAGGAAACTAATGGAA	Forward	Eurogentec
	GCCTTAAACCTTCATCTCTCAGGT	Reverse	
VP1 (CVB1)	GTAACCCTTCTTCAGGTGGAAC	Forward	Eurogentec
	GTAACCCTTCTTCAGGTGGAAC	Reverse	

Table 2. List of the primers used for quantitative RT-PCR in the present study.

Table 3. Antibodies

Antibody	Source	Company	Cat. #
α-Tubulin	Mouse	Sigma-Aldrich	T5168
β-Actin	Rabbit	Cell Signaling Technologies	4967
ALKBH5	Rabbit	Proteintech	16837-1-AP
FTO	Mouse	Invitrogen	MA5-27142
IFIT3	Rabbit	Invitrogen	PA5-22230
METTL3	Rabbit	Proteintech	15073-1-AP
MX1	Mouse	Invitrogen	MA5-31483
VP1	Rabbit	(Hovi & Roivainen, 1993)*	
OAS3	Rabbit	Invitrogen	PA5-31090
WTAP	Mouse	Proteintech	60188-1-Ig
HRP anti-rabbit IgG	Donkey	Jackson ImmunoResearch Laboratories	711-036- 152
HRP anti-mouse IgG	Donkey	Jackson ImmunoResearch Laboratories	715-036- 150

Table 3. List of antibodies used in western blot experiments in the present study.

*Hovi, T., & Roivainen, M. (1993). Peptide Antisera Targeted to a Conserved Sequence in Poliovirus Capsid Protein VP1 Cross-React Widely with Members of the Genus Enterovirus. In *JOURNAL OF CLINICAL MICROBIOLOGY* (Vol. 31, Issue 5). https://journals.asm.org/journal/jcm

Supplementary Figure 1

Supplementary Figure 1. Silencing of METTL3 in EndoC-βH1 cells. EndoC-βH1 cells were transfected with one control siRNA (siCTL) or by 3 different specific siRNAs targeting METTL3 and the combination of them. At 96h post-transfection, the protein expression was quantified by Western blot and normalized by GAPDH (A,B) and the cell viability was quantified by direct cell counting after Hoechst 33342 + propidium iodide staining (C). Results are expressed as mean +/- SEM of 6-9 experiments.

Supplementary Figure 2

Supplementary Figure 2. CVB1 induces cytopathogenic effect and viral progeny in EndoC-βH1 cells. EndoC-βH1 cells were infected with CVB1 at indicated M.O.I. (grey bars), at 24h post-infection the cytopathogenic effect was quantified by direct cell counting after Hoechst 33342 + propidium iodide staining (A) and the viral titers was measured by end-point dilution assay (TCID50/ml) (B). **** p<0,0001 vs mock; one-way ANOVA. Results are expressed as mean +/- SEM of 4 experiments.

Supplementary Figure 3

Supplementary Figure 3. Cytopathogenic Effect in iPSC – derived islets. IPSC cells were non-treated (white) or 50 µM (green) of MA and simultaneously infected with CVB1 or mock infected. The cytopathogenic effect was quantified 24h post-treatment by direct cell counting after Hoechst 33342 + propidium iodide staining.

Supplementary Figure 4

Supplementary Figure 4. Gemcitabine blocks CVB1 replication and translation in human pancreatic beta cells. EndoC-βH1 cells were treated with Gemcitabine at the indicated concentrations (A-C) or with 2 µM (D,E) at the time of infection by CVB1 at M.O.I. 0,01 (A-C), M.O.I. 1, 10 and 100 (D) or M.O.I. 10 (E). At 24h post infection, the cytopathogenic effect was quantified by direct cell counting after Hoechst 33342 + propidium iodide staining (A), the expression of VP1 mRNA was analyzed by RT-qPCR and the values were normalized by the geometric mean of β-actin and VAPA (B) and the viral titers was measured by end-point dilution assay (TCID50/ml) (C). At 6h post infection, the expression of VP1 viral protein was measured by Western blot and normalized by β-actin (D) and the expression of VP1 mRNA was analyzed by RT-qPCR and the values were normalized by the geometric mean of β-actin and VAPA (E). Results are expressed as mean +/- SEM of 2-4 experiments.
