# Supplementary figures and images for: The N6-methyladenosine RNA epigenetic modification modulates the amplification of coxsackievirus B1 in human pancreatic beta cells
Source: Front Microbiol. 2024 Dec 18;15:1501061. doi: 10.3389/fmicb.2024.1501061 (PMC11688287; doi:10.3389/fmicb.2024.1501061)

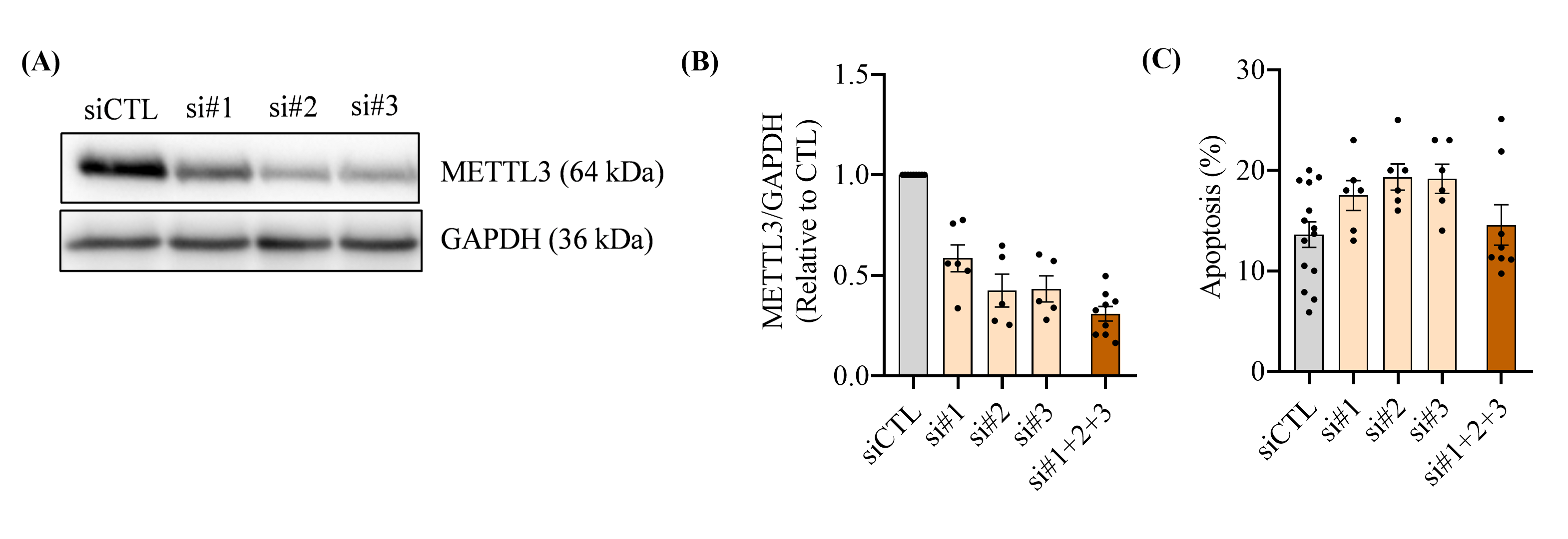

Supplement: Supplementary file 2 [file Image_1.TIF]

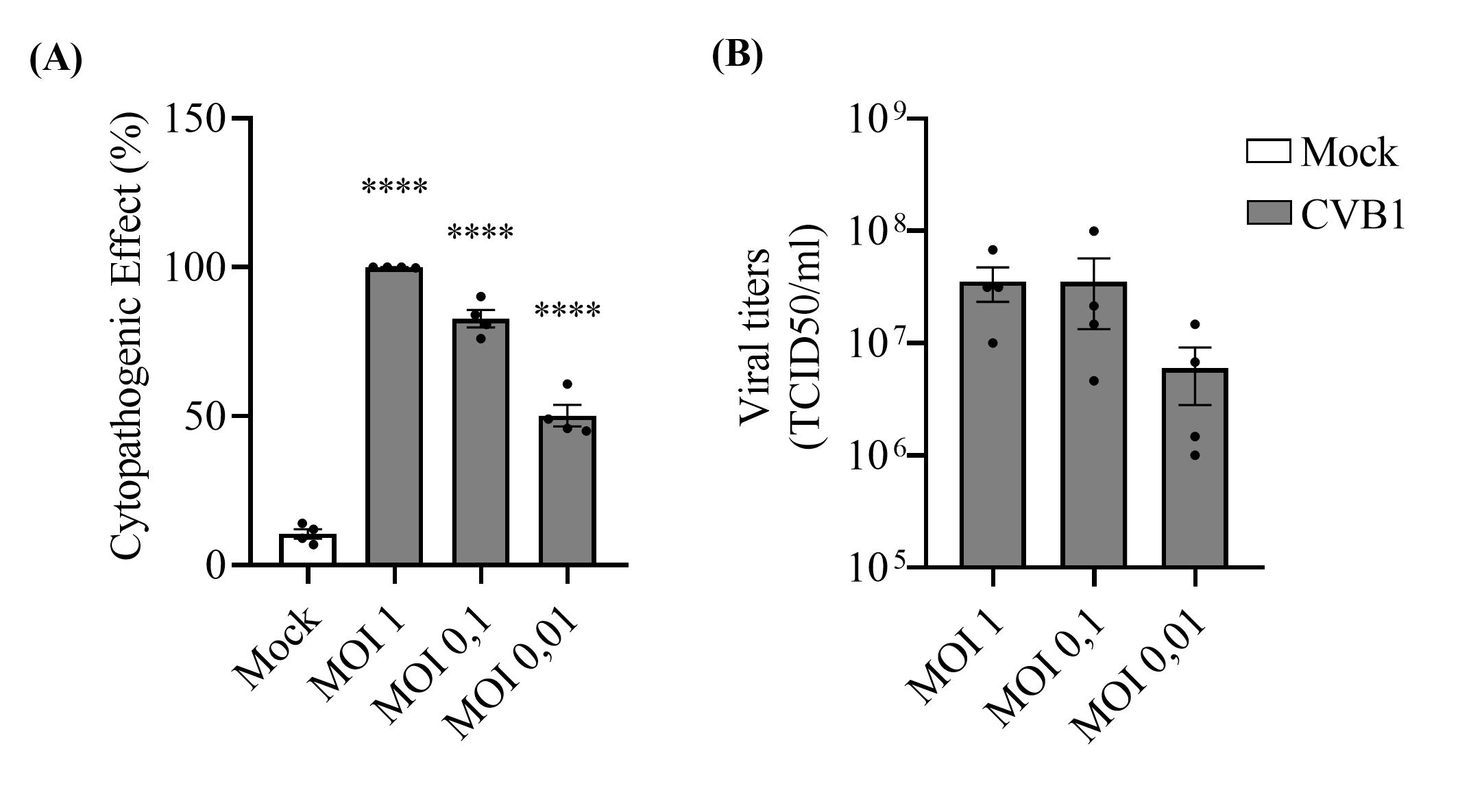

Supplement: Supplementary file 3 [file Image_2.TIF]

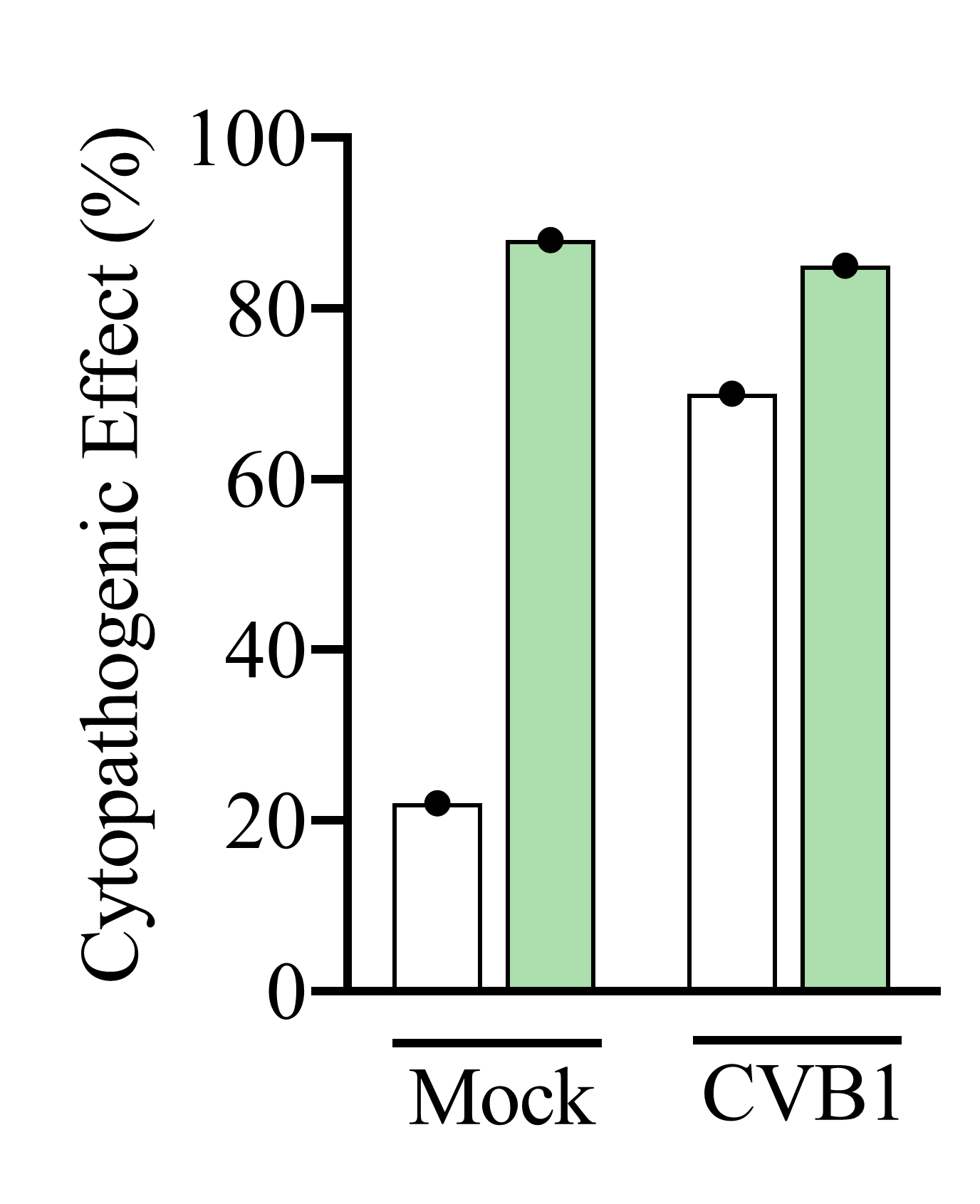

Supplement: Supplementary file 4 [file Image_3.TIF]

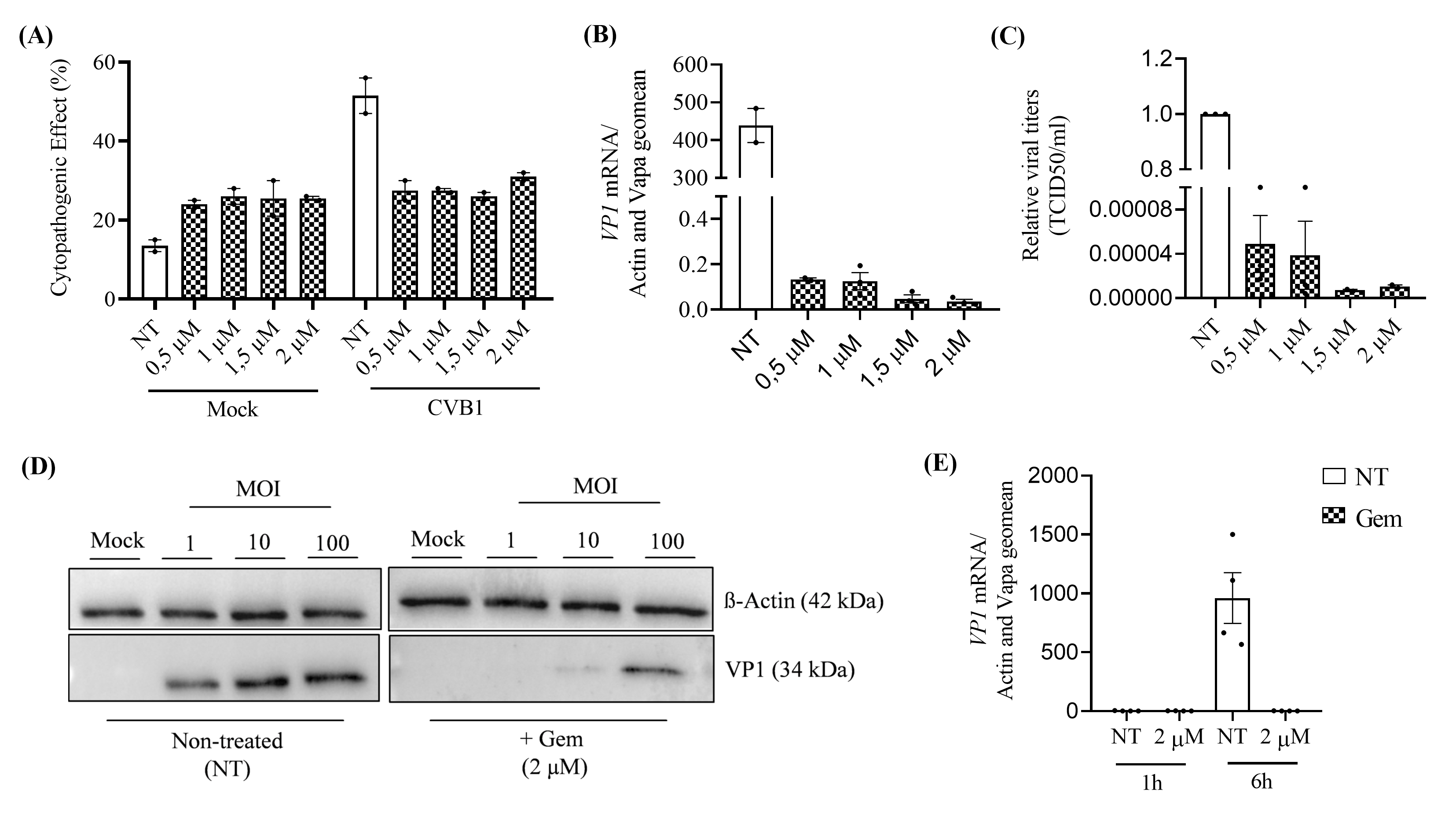

Supplement: Supplementary file 5 [file Image_4.TIF]
